# Metabarcoding analysis of the soil fungal community to aid the conservation of underexplored church forests in Ethiopia

**DOI:** 10.1038/s41598-022-08828-3

**Published:** 2022-03-21

**Authors:** Demelash Alem, Tatek Dejene, József Geml, Juan Andrés Oria-de-Rueda, Pablo Martín-Pinto

**Affiliations:** 1 Sustainable Forest Management Research Institute, University of Valladolid, Avda. Madrid 44, 34071 Palencia, Spain; 2 Ethiopian Environment and Forest Research Institute (EEFRI), P. O. Box 30708, 1000 Addis Ababa, Ethiopia; 3 MTA-EKE Lendület Environmental Microbiome Research Group, Eszterházy Károly University, Leányka u. 6, 3300 Eger, Hungary

**Keywords:** Ecology, Biodiversity, Community ecology, Conservation biology, Forest ecology, Forestry, Tropical ecology

## Abstract

Most of the Dry Afromontane forests in the northern part of Ethiopia are located around church territories and, hence, are called church forests. These forests are biodiversity islands and provide key ecosystem services to local communities. A previous study of church forest fungal species was based on sporocarp collections. However, to obtain a complete picture of the fungal community, the total fungal community present in the soil needs to be analyzed. This information is important to integrate church forests into global biodiversity conservation strategies and to understand what actions are required to conserve church forests and their biological components, including fungi, which are known for their exceptionally high diversity levels. We assessed soil fungal communities in three church forests using ITS2 rDNA metabarcoding. In total, 5152 fungal operational taxonomic units representing 16 fungal phyla were identified. Saprotrophs followed by ectomycorrhizal fungi and animal pathogens dominated fungal communities. Significant differences in diversity and richness were observed between forests. Non-metric multidimensional scaling confirmed that fungal community composition differed in each forest. The composition was influenced by climatic, edaphic, vegetation, and spatial variables. Linear relationships were found between tree basal area and the abundance of total fungi and trophic groups. Forest management strategies that consider cover, tree density, enrichment plantations of indigenous host tree species, and environmental factors would offer suitable habitats for fungal diversity, production, and function in these forest systems. The application of the baseline information obtained in this study could assist other countries with similar forest conservation issues due to deforestation and forest fragmentation.

## Introduction

Fungi occupy a wide range of ecological niches^1^. Forest soil likely represents the largest reservoir of fungi^2^. Soil fungal communities perform a variety of essential functions and, hence, play a vital role in many forest ecosystem functions^1^. Saprotrophic fungi are mainly responsible for organic matter and plant litter decomposition and thereby influence nutrient cycling^3^. Fungi provide soil carbon resources to sustain plant growth in forest ecosystems^4^. Fungal symbiotic associations with plants also enhance water and nutrient uptake by plants^5^. Soil fungal pathogens influence the species diversity, composition, dynamics, and productivity of plant communities^6,7^. Furthermore, fungi serve as symbols of conservation and indicate the health of most terrestrial ecosystems^8,9^, including forest ecosystems^2^. However, the vegetation usually determines the limit of fungal ranges^10,11^. As a habitat, the forest soil environment also impacts fungal communities^12^. These impacts differ with climate, topography, vegetation type, and the magnitude of disturbances in the forest^13^. Thus, given their role in the ecosystem, it is imperative to study fungi in different ecosystems to gain a rigorous understanding of how fungi respond to ecosystem properties to set up appropriate management and conservation strategies.

The Ethiopian highlands are the largest Afromontane region in Africa^14^. Many biodiversity hotspots are known to exist in the Ethiopian Afromontane forests^15^. However, Afromontane forests are one of the most fragmented ecosystems, particularly in the Northern part of Ethiopia because of forests land degradation and deforestation due to anthropogenic processes, and their conservation is a high priority in the country^16^. These forest fragments, which are generally located around church territories and, hence, are called church forests, have survived because of cultural or religious values held by local communities, which have contributed to the conservation of their biodiversity^17–19^. The local people also rely on these church forests for the provisioning of livestock feed, tree seedlings, fuelwood, honey, clean water, and other essential ecosystem services including shade, climate regulation, habitat for pollinators, and spiritual values^18^. Although, the church forests vary in their size, and shape, Wassie et al.^20^ reported up to 100 ha of forests lands. However, this value may increase depending on the area. Owing to the small size of these forest fragments, there are variations in their biodiversity^21^ because fragmentation can result in variations in species richness and composition^16^. These variations might also be shown in the patterns of associated biological resources in several ways, including microhabitat change and habitat isolation^22^. Furthermore, fragmentation results in limitations that affect the population viability in the long-term^22^. Several studies have evaluated the conservation value of the fragmented Dry Afromontane church forests in the Northern landscapes of Ethiopia^15,16,18^. However, to facilitate the conservation of economically and ecologically important species in these forest systems, the ecology and conservation status of the fungi inhabiting these forests needs to be determined.

Recently, there has been an interest in surveying fungi in particular habitats^22^, to describe and predict the extent of their diversity at a larger scale^23^. This information is important to enable the integration of fragmented Afromontane church forests into global biodiversity conservation strategies^15,18^ and to understand what actions are required to conserve fragmented forests and their biological components, including fungi, which are known for their exceptionally high diversity levels^24^. However, only a few studies have assessed the fungal diversity of the Dry Afromontane regions of Ethiopia^17,25^. These studies focused on macrofungi inhabiting a few of the forests in this region. Větrovský et al.^26^ suggested that a metabarcoding analysis of the composition of fungal communities in individual studies could be used to map the global diversity of soil fungi. Given that there has been little investigation of the fungal diversity in tropical regions^27^, this type of study could also help to explain tropical fungal ecology and diversity in the global distribution of soil fungi and also contribute to the GlobalFungi database^26–29^. However, there is a consensus that fragmentation has an impact on soil properties and the belowground soil organisms^30–33^. Variation in environmental variables such as temperature^34^, precipitation^27^, altitude^35^, soil pH^36^, nutrient availability^37^, and the plant community^38^ also influence fungal diversity and the community composition. Thus, exploring how environmental variables affect fungal communities may be meaningful for managing fragmented church forests and their ecosystem components^39^. However, as yet, information on how habitat fragmentation and the environmental variables may affect fungal communities or limit fungal processes is relatively limited^25,40,41^. The overall objective of this study was to provide scientific insight into the soil fungal communities of fragmented church forests in Dry Afromontane regions of Northern Ethiopia to promote the conservation of these valuable forests. Thus, in this study, we evaluated the soil fungal diversity and community composition associated with these relict ecosystems by sampling soils of church forests in Northern Ethiopia. Despite fragmentation, church forests are rich in plant species diversity^18^. Fungal diversity is related to vegetation characteristics^42^. A previous study based on sporocarp collections in these forests reported that macrofungal sporocarp composition differs among church forests^17^. In order to obtain further information on the fungal diversity and composition in this type of forest, this partial knowledge based on taxa that are able to fruit needs to be supported and complemented with a deeper analysis of the fungal community present in the soil of church forests. Accordingly, we expect that in this study, the soil fungal community will differ among church forests, resulting in overall higher richness and diversity values^17^. Also, the fungal diversity will be driven by site conditions such as climate and soil fertility^27,43^. Thus, our specific aims were to study three church forests in Dry Afromontane areas of Northern Ethiopia: (1) to describe fungal operational taxonomic unit (OTU) richness and diversity; and (2) to determine whether and how the soil fungal community composition was governed by the spatial characteristics of the church forests, including vegetation, climate, and soil variability.

## Results

### Fungal OTU taxonomic composition

From the total amplifiable fungal diversity, a total of 1,842,446 high-quality sequences were obtained, with a mean of 68,238.7 reads and a minimum of 34,141 reads in each sample, representing 5152 fungal OTUs and 16 fungal phyla. Overall, Ascomycota dominated the fungal community (2332 OTUs) followed by the Basidiomycota, which together accounted for 71.48% of the sequences. The ranking of taxonomic orders belonging to Ascomycota, based on the number of representative OTUs, was as follows Hypocreales (375), Pleosporales (241), Chaetothyriales (230), Eurotiales (179), Helotiales (123), and Sordariales (109), followed by many other orders with less than 100 fungal OTUs (Table S1). Agaricales (524) and Thelephorales (353) were the most species-rich orders of Basidiomycota, followed by other orders with less than 70 OTUs each. Unidentified fungi were classified down to kingdom level and represented 780 OTUs; 15.14% of the total. Several species belonging to the *Agaricus*, *Boletus*, *Geastrum*, *Lepiota*, *Psathyrella*, *Russula*, *Termitomyces*, *Tomentella*, or *Trichoderma* genera were also identified with at least 98% similarity with the reference sequences. Surprisingly, some of these species are ectomycorrhizal fungi, even though the studied forests are considered to be non-ectomycorrhizal ecosystems. The taxonomic phylum, order, and genus of fungal OTUs are listed in Table S1.

**Figure 1:**
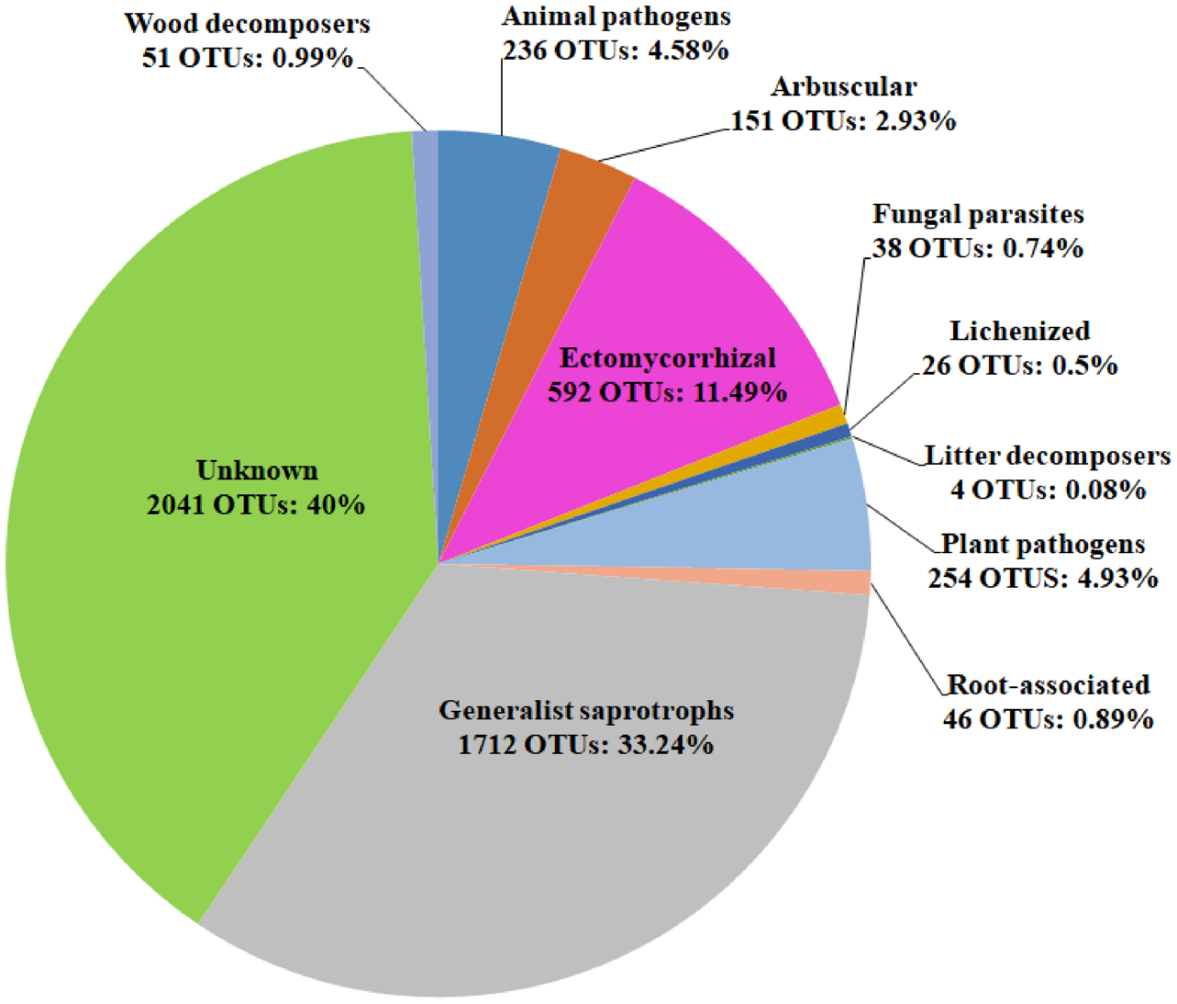
Relative proportions of fungal operational taxonomic units (OTUs) according to their guild assignment at the genus level (trophic groups; the number of OTU; percentage).

**Figure 2:**
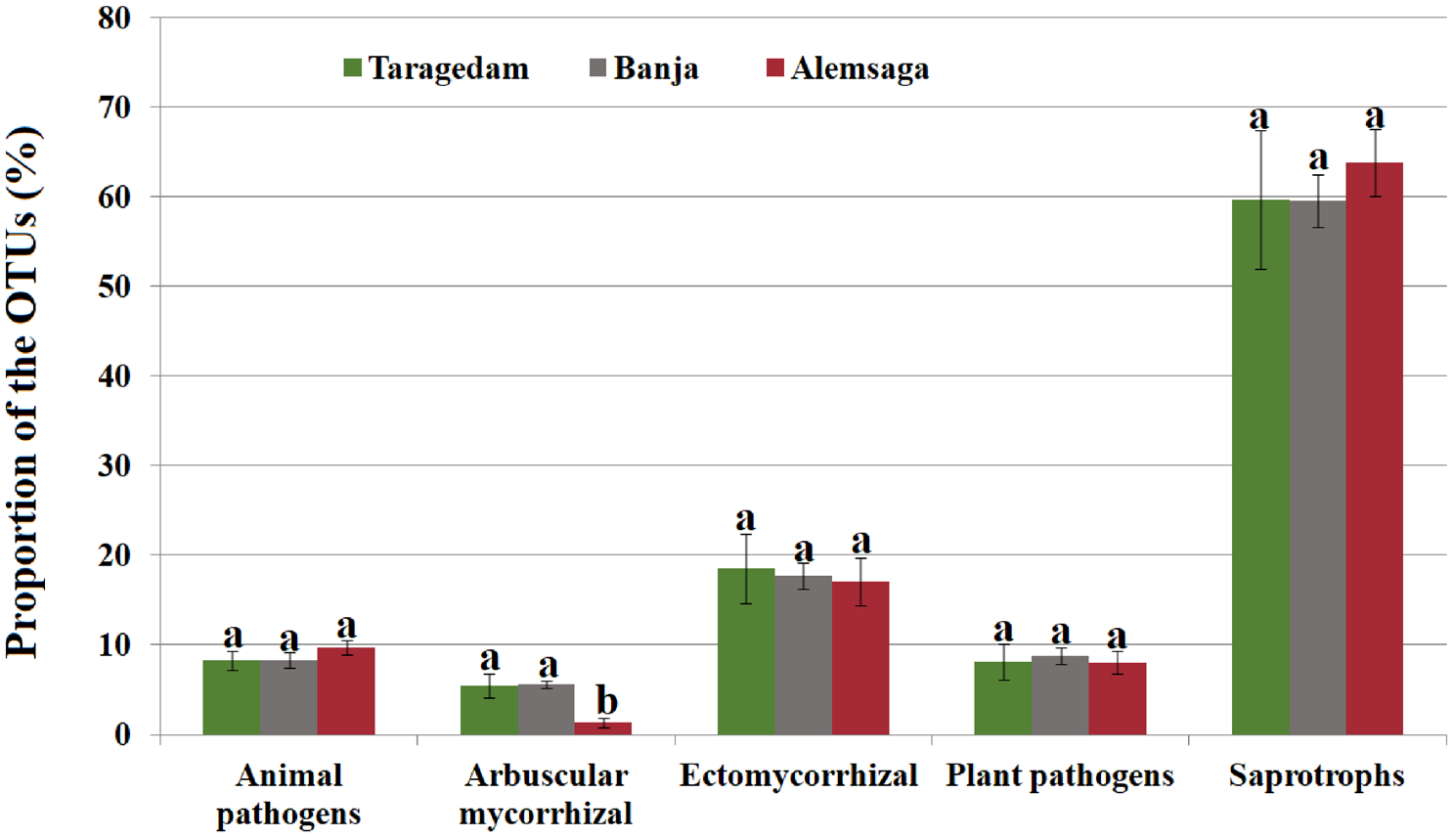
The relative richness of the fungal operational taxonomic units (OTUs) of main fungal trophic groups among the three studied Dry Afromontane church forests. Values with the same letter in a group are not significantly different. Bars denote the standard error.

Fungal OTUs were further assigned to trophic groups (Fig. 1). Overall, saprotrophs were the most abundant guild across the whole dataset, representing 33.24% of the community abundance, followed by ectomycorrhizal fungi (11.49%), animal pathogens (4.58%), plant pathogens (4.93%), and arbuscular mycorrhizal (2.93%). Less dominant groups were represented by wood and litter decomposers, root-associated fungi, lichenized fungi, and fungal parasites. About 40% (2041) of the fungal OTUs were not assigned to a trophic group. Only the arbuscular mycorrhizal showed significant differences among the three church forests (F = 16.16; *p* = 0.0004; Fig. 2). There was no significant difference when comparing the relative richness of the ectomycorrhizal, saprotrophs, plant pathogens, and animal pathogens among the three church forests (*p* > 0.05; Fig. 2).

### Soil fungal richness and diversity

**Figure 3:**
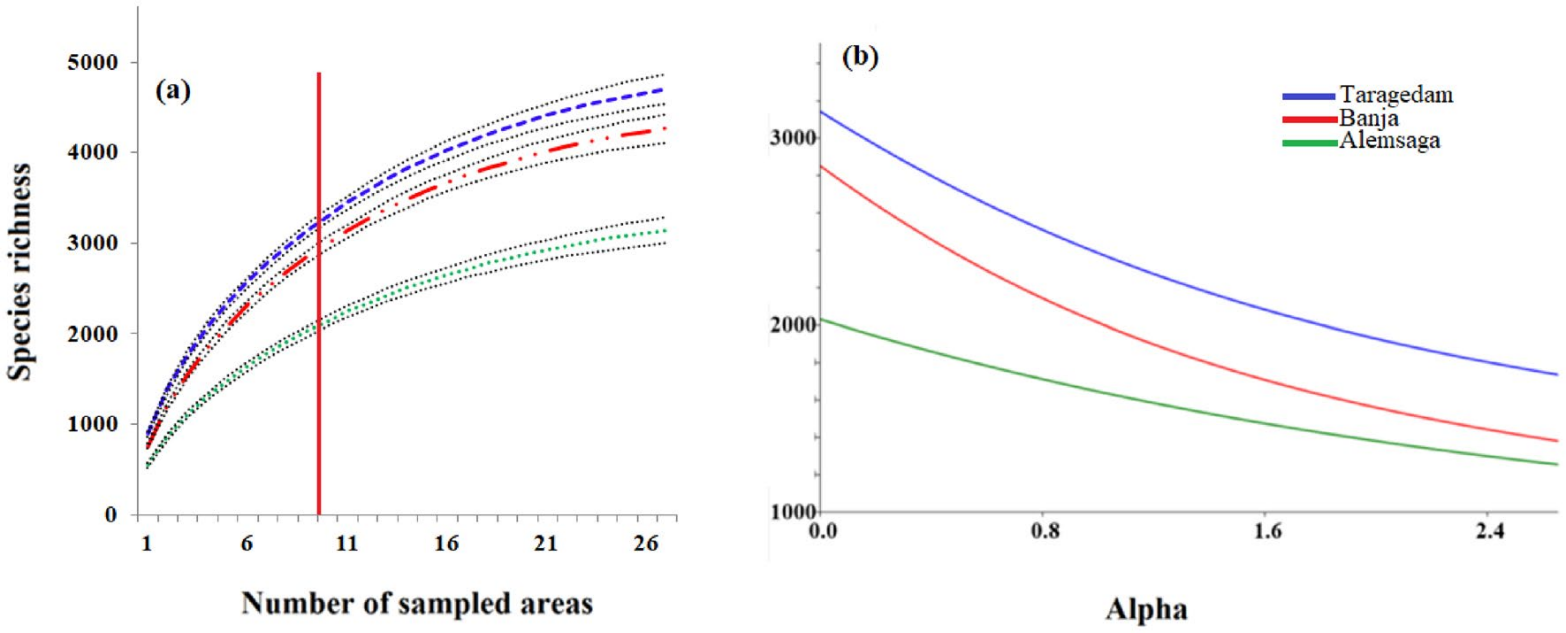
Patterns of fungal species were recorded in three Dry Afromontane forests in Northern Ethiopia. (**a**) Observed species accumulation curves across the fragmented forests using the rarefaction sample-based estimator of EstimateS. Data were extrapolated following procedures proposed in the EstimateS manual^44^, the points to the right of the red vertical line are the curve that is extrapolated, (**b**) Rényi diversity profiles of log-transformed fungal OTU abundance data. The profile values for alpha = 0, 1, 2, and infinity indicate the species richness, Shannon diversity index, the logarithm of the reciprocal Simpson diversity index, and Berger Parker diversity index, respectively. If the profile for one sample was consistently higher than the profile for another sample, the sample with the higher-profile was considered more diverse. When curves for two communities intersect, this means that they cannot be ranked. Lines represent the species richness or diversity of the forests. The black lines are the 95% confidence intervals of each curve.

The overall accumulation curves for the three forests showed that fungal richness saturation was not reached, with the curves showing a steady increase even after data extrapolation (Fig. 3a). However, a significant difference in the overall OTU richness was observed among the three church forests. The Taragedam forest showed the highest level of richness, and this value was significantly different from those of the Banja (*p* = 0.010) and Alemsaga (*p* = 0.000) forests. The fungal OTU richness of the Banja and Alemsaga forests also differed significantly (*p* = 0.000). Shannon’s H’ fungal diversity also showed a distinct trend among forests. The highest value (5.31 ± 0.30) was recorded for the Taragedam forest followed by the Banja (4.73 ± 0.25) and the Alemsaga (2.37 ± 0.08) forests, indicating that the Alemsaga forest was significantly different from the Taragedam (*p* = 0.001) and Banja (*p* = 0.010) forests. The diversity profiles of the Taragedam and Banja forests are more asymptote and their diversity values do not differ significantly (Fig. 3b; *p* = 0.182).

Significant differences were found between the three forests when comparing tree parameters, such as basal area and tree canopy cover values (Table 4). In addition to these differences, when analyzing trophic groups, linear relationships were found between basal area and the abundance of total fungi (r = 0.42, *p* < 0.05), arbuscular mycorrhizal fungi (r = 0.50, *p* < 0.01), and ectomycorrhizal fungi (r = 0.04, *p* < 0.05). Similarly, the Shannon diversity value of the total fungal species, animal pathogen species, ectomycorrhizal species, and fungal parasites were also significantly correlated with tree canopy cover (*p* < 0.05). In addition, tree canopy cover correlated with total fungal richness, animal pathogen richness, and ectomycorrhizal species richness (*p* < 0.05). r values indicating the correlation between fungal and vegetation parameters and their level of significance are provided in Table 1.

**Table 1:** Correlation of vascular tree parameters and fungal variables plus the corresponding significant *p*-values.

Tree parameters	Fungal parameters	r	*p*-values
Tree basal area	The abundance of total fungal species	0.42	*p* = 0.05
	The abundance of arbuscular mycorrhizal species	0.50	*p* = 0.01
	Abundance of ectomycorrhizal species	0.40	*p* = 0.05
Tree canopy cover	Shannon diversity value of total fungal species	0.46	*p* = 0.05
	Shannon diversity value of animal pathogens	0.42	*p* = 0.05
	Shannon diversity value of ectomycorrhizal fungi	0.40	*p* = 0.05
	Shannon diversity value of fungal parasite species	0.38	*p* = 0.05
	Richness of total fungal species	0.40	*p* = 0.05
	Richness of animal pathogen species	0.45	*p* = 0.05
	Richness of ectomycorrhizal fungal species	0.39	*p* = 0.05

### Soil fungal composition and environmental variables

**Figure 4:**
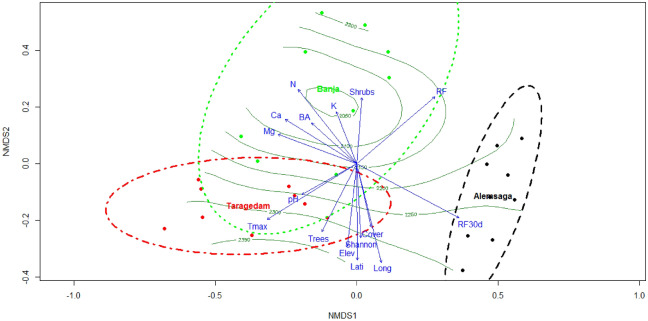
Non-metric multidimensional scaling (NMDS) ordination graph with fitted environmental variables based on dissimilarities calculated using the Bray–Curtis index of fungal community compositions of the three Dry Afromontane church forests in Northern Ethiopia with vascular tree richness displayed as isolines. Arrows represent environmental variables that were most significantly (*p* ≤ 0.005) related to ordination. Ellipses indicate forest groups. The explanatory variables are shown in blue. RF, annual rainfall; Tmax, average daily maximum temperature for 2019; BA, tree basal area; RF30d, cumulative rainfall 30 days before sampling; Elev, elevation a.s.l.; Lati, latitude; Long, longitude.

The NMDS based on Bray–Curtis distance followed by the perMANOVA analyses confirmed that soil fungal communities differed among the three church forests (F = 2.64, R^2^ = 0.19, *p* = 0.010). The same result was obtained when the NMDS was checked by using present and absent data analysis (F = 4.47, R^2^ = 0.27, *p* = 0.001). However, the ordination pattern showed that the soil fungal community composition of Alemsaga forest is more distinct than the communities in the other two forests are from each other (Figs. 4; S1). ANOSIM pair-wise comparisons measuring the strength of the differences in fungal composition between the three church forests are shown in Table 2.

**Table 2:** ANOSIM pair-wise comparisons of soil fungal composition between the three fragmented forests based on Bray–Curtis distance measures (Global R-value = 0.75; *p* = 0.001).

Pair-wise comparison between forests	R-value	*p-*value
Alemsaga and Banja forests	0.86	0.0002
Alemsaga and Taragedam forests	0.79	0.0001
Banja and Taragedam forests	0.58	0.0001

Analysis of the influence of environmental variation revealed that explanatory environmental variables grouped under spatial, climate, vegetation, and edaphic parameters were also significantly correlated with the soil fungal community composition (*p* < 0.050; Fig. 4; Table 3). Specifically, individual spatial and climatic parameters showed a highly significant influence on the composition of soil fungi in church forests (Table 3). Similarly, among the vegetation and soil parameters, tree density and N parameters strongly influenced the fungal community structures. Isolines shown on the NMDS ordination (Fig. 4) represent vascular tree richness values across the three studied forests.

**Table 3:** Significance of explanatory variables for soil fungal community composition based on the Hellinger transformed matrix. Numbers in bold indicate a highly significant effect (*p* < 0.001). Grouped contribution is shown according to the Mantel test.

Sources	Contribution	Variables	pseudo-F	*p*
Soil fertility	30.16%	pH	0.2736	0.021
		Ca	0.4626	0.002
		Mg	0.4787	0.002
		K	0.2332	0.047
		N	0.5459	**0.001**
Climate	62.87%	Annual rainfall	0.7019	**0.001**
		RF30d	0.8736	**0.001**
		Average daily max temperature	0.7448	**0.001**
Spatial factors	6.17%	Elevation	0.4110	0.002
		Latitude	0.5126	**0.001**
		Longitude	0.5805	**0.001**
Vegetation	16.91%	Tree density	0.3887	**0.001**
		Shrub’s density	0.3027	0.004
		Cover	0.2899	0.011
		Basal area	0.2319	0.046
		Tree diversity	0.3149	0.007

## Discussion

### Fungal OTUs taxonomic composition

In the highland regions of Northern Ethiopia, patches of Dry Afromontane forests around churches and inaccessible mountain areas are a reservoir of biodiversity^15,18^. However, to manage this kind of forest appropriately, a quantitative and objective assessment and description of their biodiversity components need to be undertaken^45^. In particular, knowledge of fungi inhabiting these forests is vital because fungi interact with plants as pathogens or mutualists, or are involved in recycling organic matter^27^. Although the PCR is known to introduce biases by skewing the amplification profile concerning GC content^46^, in this analysis, we systematically utilize PCR reactions of each sample by carrying out in triplicate to minimize the amplification bias and to improve PCR success rates. Thus, a total of 5152 fungal operational taxonomic units (OTUs) representing 16 fungal phyla could be retrieved from the analysis. A considerable number of important fungi were observed from the result. Many of these species are saprotrophs, which are ecologically important and are also valuable as a source of food for humans^47^. Some species can also be used in agriculture as biocontrol agents^48,49^. Most of the species detected in the genera *Agaricus* and *Termitomyces* have been reported previously as fruit bodies from the southern part of Ethiopia^50^ and are used as food by local communities in the country^51^. Interestingly, the *Termitomyces* species are usually collected from the fields and can be used as seasonal food in some localities in Ethiopia during periods of food shortage^52^. In addition, several species belonging to the genera *Boletus*, *Russula*, or *Trichoderma* that form symbiotic associations with vascular plants were also detected^11^. Some species of *Boletus* and *Russula* are known to produce edible sporocarps, which, in some cases, are highly appreciated and prized all over the world. On the other hand, a number of fungi were unidentified due to the incomplete taxonomic classification of the matching reference sequences in the database, mostly because of taxonomic uncertainties of the matching fungi^53^. However, the result exhibited the occurrence and distribution of different fungal guilds at a general level, which can help us to generate hypotheses and guide for future studies in the country. In light of this, the present study provides important information that contributes to our knowledge of Ethiopian fungal diversity^54^. Indeed, we also appreciate the limitation of comparing the proportional distribution of different fungal taxa across the three studied forests. Thus, further research to address the above issues will help build on our study to produce a more comprehensive picture of the structure and functioning of fungal communities in this region.

### Soil fungal richness and diversity

We observed variation in the overall soil fungal richness and diversity among the three studied forests. The Taragedam forest had the highest richness and diversity values, followed by the Banja and the Alemsaga forests. This pattern of fungal diversity may reflect a difference in ecological features^55^ or plant species diversity among the studied forests^56^. When considering the dominant trophic groups separately, the proportional distribution of root-associated fungi such as arbuscular mycorrhizal guilds differed significantly between the three forests. The higher arbuscular fungal OTUs were observed in the Taragedam and Banja forests. These fungi are vital contributors to ecosystem functioning, as the trees' nutrient acquisition strategies involving arbuscular mycorrhizal associations are key plant functional traits leading to distinct carbon (C) and nutrient dynamics in forests^51^. Furthermore, the arbuscular mycorrhizal fungi are reported dominant in the organic soil of the forests^57^. Interestingly, in our analysis, the higher organic matter was obtained from the Taragedam and Banja forests where we got a higher proportion of arbuscular mycorrhizal species. Although the factors that determine their assembly are still poorly understood^58^, the high proportion of arbuscular fungi in the two forests might also be explained in terms of the associated relative availability of a broad host range in the studied forests^17^. Hence, there may be more trees that can act as hosts for arbuscular mycorrhizal fungi^17^. Thus, our findings present an insight into the conservation and management of valuable trophic groups in the soils of fragmented Dry Afromontane church forest systems in Ethiopia.

### Soil fungal community composition and environmental variables

Evaluating fungal communities in different ecosystems is essential to filter out the relative contributions of environmental factors to fungal composition in an ecosystem^59^. In this study, although the NMDS showed the fungal species composition changes among forests, the proportion of the different functional guilds looks quite consistent at the forest level. The observed compositional difference may be explained by site-level differences in the climate and edaphic factors, that are known to influence fungal structures in forest systems^60^. The differences in the fungal communities among the three studied forests underlines the conservational value of each particular fragment of the church forest. For example, the Alemsaga church forest has a distinctive fungal community composed of mycorrhizal species in the genera of *Tomentella*, *Piloderma*, *Byssocorticium*, and *Cortinarius*. Of these, the genus *Tomentella* is frequently found in coniferous and deciduous forests worldwide^61^. However, the existence of these ectomycorrhizal species in the Alemsaga church forest may be due to the dispersion of mycorrhizal inocula from nearby plantation forests, which are dominated by *Eucalyptus* and *Pinus* species^62^, which are ectomycorrhizal-associated. This finding may be an indicator of the ecological restoration status of the trophic structure of the soil fungal community^55^. Furthermore, the findings presented here may have important implications for the maintenance of functional guild diversity in the indigenous forest system given that mycorrhizal fungi have previously only been reported from exotic tree plantations^50^. Further empirical studies are needed to confirm the associations of mycorrhizal species with indigenous forest systems in Ethiopia, as ectomycorrhizae are not typically parts of these forests systems.

The fungal composition of the Taragedam forest is also characterized by generalist saprotrophs species. In this case, climatic variables such as mean annual precipitation and temperature could be important factors driving this composition, in addition to other variables such as shrub cover, tree density, and soil variables^63^. This is because the Taragedam forest is relatively warm with a mean annual temperature of 19.5 °C, lower humidity than the other two church forests, and mean annual precipitation of 1098 mm. The Taragedam forest also has a higher relative diversity of tree species and less shrub cover than the other forests, thus there might be more fungal species associated with this specific condition, resulting in the formation of a distinct community. The analysis also indicated that non-litter decomposer species such as *Ramicandelaber*, *Polyschema*, *Mortierella*, *Calocybe*, *Auxarthron*, *Trichoglossum*, and *Geoglossum* are more favored in the Taragedam forest. Among the listed species, the abundance of *Polyschema* and *Trichoglossum* species were reported greater in forests with higher temperatures^64,65^. This may be explained by the microclimate conditions of forest ecosystems^66^, particularly temperature^67^ that the temperature in the Taragedam forest may have been hospitable to many non-litter decomposer fungi as soil microbial activity rises exponentially with soil temperature^68^. Furthermore, as the fungal community structure is influenced by their feeding patterns^69^ in forest systems, the plant species composition may also influence fungal community structure due to the low availability of decomposable material on the forest floor of the study areas, and thus, non-litter decomposers would be promoted in these systems, as non-hospitable conditions could shift the abundance or structure of microbial communities in the forest soil^70,71^. Although Banja forests had lower species richness, it was also dominated by non-litter decomposers belonging to the genera *Entoloma*, *Rosasphaeria*, *Boubovia*, and *Omphalotus*. A possible explanation for this could be due to the available organic matter in the soil, which could favor such kinds of species compositions because organic matter amendments and moisture availability generally enhance the diversity and composition of non-litter fungi in the soil^72^.

It is well established that fungal communities as a whole are significantly influenced by edaphic variables. This is because the soil contains nutrients that enable fungi to grow and develop^73^ and, thus, in turn, the composition of fungi is directly influenced by the soil fertility of a site^73,74^. The pH and N of our study sites were correlated with the composition of the soil fungal community together with other cation elements, such as Ca, Mg, and K. Among these, pH and N are considered to be the most influential parameters governing fungal composition^75^. Our forest soil samples were relatively acidic, with pH values ranging between 5.60 and 7.04 (the mean pH calculated from the [H +]), indicating that the fungal communities that inhabit these soils can tolerate a low pH level. Low soil pH conditions may affect the fungal community composition through its influence on spore germination and mycelial development^36^. In addition, soil pH influences the availability of carbon and nutrients in the soil on which fungal growth and biomass composition are dependent^76^. We also found that some fungal taxa are able to tolerate a higher pH level (for example in the Taragedam forest). One of the reasons for this might be that these taxa characteristically are adapted to and grow in comparatively alkaline soil, resulting in an increase in the community richness without inhibition of their growth^59^, as some fungi can also grow well in neutral to slightly alkaline conditions^77^. Previous studies have suggested that N could affect microbial community structures depending on the forest type^78^. This is because the soil N availability negatively influences fungal growth, particularly of mycorrhizal fungi, and has a significant effect on community composition^79^. In this study, we observed an obvious correlation of N with the entire fungal community composition of the three church forests. A relatively higher N value was estimated for the Banja Forest as compared to the other two forests. This might be an indication that the majority of plant species in the studied forests have not formed an association with mycorrhizal fungi. A high level of N availability in the soil could reduce the dependency of the host plant on fungi, which eventually could cause competition among the fungal species and could lead to distinct community compositions^79^. Cations can also play an important part in many physicochemical processes, such as photosynthesis^80^, and, thus, can affect plant photosynthesis and, hence, the amount of carbon that is available to soil fungi^81^ in forest soils.

## Conclusion

This study was carried out in relatively natural forests in a region where fungi had not been previously studied using metabarcoding and where cultural attitudes to church forests provide the potential for conservation. Thus, this work in a little-explored biogeographic region could provide valuable scientific insight to promote the conservation value of these remaining forests. Also, given that there has been little investigation of the fungal diversity in tropical regions, the information could be used to map the global diversity of soil fungi from the tropical forests system as well. Due to the key ecological role that fungi play in ecosystem functioning, information about how ecological factors affect fungal communities in these fragmented church forests could be crucial to enable the integration of these forests into global biodiversity conservation strategies and to understand what actions must be undertaken to conserve these forests, and their biological components. Thus, we investigated the diversity and community composition of soil fungi to suggest management and conservation strategies for church forests through identifying crucial roles played by fungi in the management and protection of these forest systems. Depending on the forest type, we identified various valuable fungal species belonging to the genera of *Tomentella*, *Piloderma*, *Byssocorticium*, and *Cortinarius*. Furthermore, we identified the dominancy of guilds such as generalist saprotrophs in all the forests, reflecting their similarity in terms of tree richness and diversity, which might also lead to unique soil fungal diversity and activity due to greater niche availability as a result of carbon sources, root exudates in the soil, variable microclimate, and the spatial and age variability of trees. Unsurprisingly, the soil fungal communities as a whole were also influenced by climatic and edaphic variables given that both these variables can influence mycelial development in the soil. The separate analysis of the vegetation characteristics also showed that the fungal community can be affected by differences in plant community variables. Some of the vegetation parameters such as tree cover and basal areas were also found to be positively associated with the trophic groups. The conservation and management of fungi in these forest systems could be maintained through forest management because they can modify vegetation parameters such as tree density, canopy cover, basal area, understory plant communities and, thus, could play a crucial part in shaping fungal communities. Furthermore, the promotion of native host diversity by taking into consideration the suitability of the climate and the spatial characteristics of an area through enrichment plantation systems would offer suitable habitats with variable microclimates to support diverse compositions of fungi in fragmented forest systems. The management of church forests is necessary because the conservation of fungi could assist with the successful rehabilitation of these fragmented forests as fungi have direct consequences on plant growth through mutualism, pathogenicity, and their effect on nutrient availability and cycling, as well as playing vital roles in soil organic matter stabilization and the decomposition of residues.

## Methodology

### Description of the study areas

**Figure 5:**
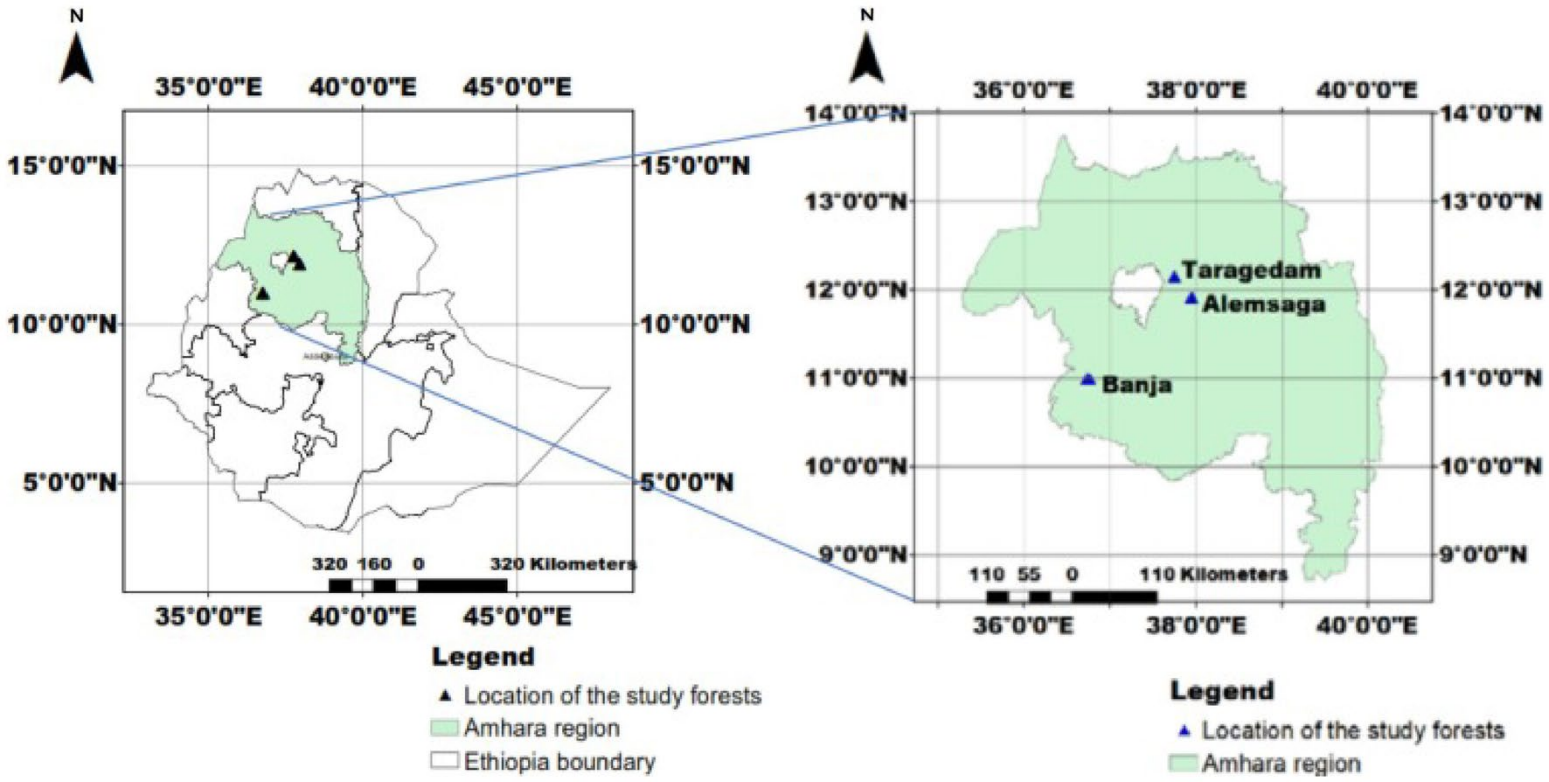
Map of the Amhara region in Northern Ethiopia showing the location of the three church forests in which the study plots were located.

Three church forests were selected to evaluate the fungal communities in a set of plots that were randomly distributed in each of the church forest areas: namely the Taragedam forest located in *Libokemkem* district, the Alemsaga forest located in *Farta* district, and the Banja forest located in *Banja* district of the Amhara region, Northern Ethiopia^82^ (Fig. 5). These forests are fragments of the remnant Dry Afromontane forests in Northern Ethiopia^82^. The Taragedam and the Banja forests were designated as reserve forests in 1979^83^ and 1994^84^, respectively, to prevent any kind of encroachments. The Alemsaga forest was designated as a priority forest in 1978 to serve as a seed source, to conserve the remnant natural forest, and to rehabilitate the degraded area in the Northern part of the country^85^.

### Soil sampling for molecular and edaphic analysis

Within each of the church forests, nine 2 × 50 m plots were sampled in 2019. They were established randomly, generating random points on a map and using these as plot locations. The plots were laid out at least 500 m apart to avoid confounding spatial effects inherent to such a plot-based design^86^. Plots were analyzed as independent samples^3^. In each plot, five soil cores were extracted 5 m apart along the centerline of each plot using a cylindrical soil borer (2 cm radius, 20 cm deep, and 250 cm^3^)^87^ to collect samples with spatial variability while minimizing the likelihood of repeatedly sampling the same genet. Litter and twigs were removed from the surface before soil cores were taken^88^. The five cores from each transect were pooled to produce a composite soil sample for each plot established in each forest. The samples were transported to the laboratory in sterile plastic bags and stored at 4 °C. Samples were frozen immediately upon return to the laboratory and kept at − 20 °C until DNA was extracted. Next, the samples were dried, sieved through a 1-mm^2^ mesh, and then ground to a fine powder using a mortar and pestle. Each sample was subjected to genomic DNA analysis, which was performed using 100 g of soil, and physicochemical analysis, which was performed using two 20 g samples of each soil sample. We maintained care in fieldwork and lab work to avoid cross-contamination among samples as much as possible, e.g., sampling tools were cleaned with alcohol (96%) after sampling each plot.

The physicochemical analysis of the soil samples was performed by the Amhara Design and Supervision Works Enterprise at Bahir Dar, Ethiopia, following standard extraction methods (i.e., diethylene triamine pentaacetic acid extraction, KH_2_PO_4_ extraction, Olsen, Kjeldahl digestion, Walkley–Black, and ammonium acetate) and using instrumental analysis to determine the pH, organic matter, cation exchange capacity, sodium (Na), potassium (K), calcium (Ca), magnesium (Mg), nitrogen (N), available phosphorus (P), and physical properties (% of sand, silt, and clay) of the soil. Soil pH and electrical conductivity were determined by analyzing a soil:water (1:2.5) suspension and the supernatant from the same suspension with the aid of a pH meter and an electrical conductivity meter, respectively^89^. The organic carbon content of the soil was determined using wet digestion^90^. The Kjeldahl procedure was used to determine the total N content in soil^91^. Sodium bicarbonate (0.5 M NaHCO_3_) was used as an extraction solution to determine the available P^92^. Available Na, available K, Ca, and Mg were also determined. To assess soil particle size we used a hydrometer^93^ and sodium hexametaphosphate (Calgon solution) was used as a dispersing agent. After calculating the proportions of sand, silt, and clay, the soil was assigned a textural class name using ASTM free software, Version 4 (http://www.astm.org). Comprehensive descriptions of the forests and the soil variable analyzed from each of the church forests are provided in Table 4.

**Table 4:** Comprehensive descriptions and selected edaphic variables of the three church forests, Northern Ethiopia.

Descriptions	Forests		
	Taragedam	Alemsaga	Banja
Geographical location	12°06'–12°07' N 37°46'–37°47' E	11°54'–11°56'N 37°55'–37°57'E	10°57'–11° 03'N 36°39'–36°48'E
Altitude range (m asl)	2142–2484	2180–2470	1870–2570
Mean annual precipitation (mm)	1098	1926	1884.3
Mean annual temperature (°C)	19.5	15.8	18.7
CRF30d (mm)	1488	1926	1884
Forest area (ha)	875	814	897
Density of trees ha^–1^	48.11	17.19	43.13
Canopy cover (%)	800.9 ± 7.02a	82.18 ± 2.16a	67.49 ± 6.16b
Basal area m^2^	4.95a	1.72b	4.36a
Shrub density ha^–1^	146a	747b	2881b
Tree Shannon values	2.18 ± 0.08a	2.04 ± 0.07a	1.38 ± 0.12b
Sand (%)	58.89 ± (2.93)b	51.78 ± (2.99)b	68.67 ± (2.21)a
Silt (%)	28.44 ± (2.38)a	32.44 ± (2.13)a	20.00 ± (1.76)b
Clay (%)	12.67 ± (1.37)a	15.78 ± (1.93)a	11.33 ± (1.33)a
pH H_2_O 1:2.5	7.04 ± (7.03)a	5.85 ± (6.59)b	5.60 ± (6.24)c
EC (dS/m)	0.43 ± (0.05)b	0.28 ± (0.03)b	0.81 ± (0.14)a
Ex.Ca (cmol( +)/kg)	13.95 ± (0.60)a	9.19 ± (0.52)b	13.55 ± (0.87)a
Ex.Mg (cmol( +)/kg)	6.16 ± (0.10)a	4.58 ± (0.15)c	5.54 ± (0.20)b
Ex.Na (cmol( +)/kg)	1.95 ± (0.05)a	2.05 ± (0.10)a	1.82 ± (0.12)a
Ex.K (cmol( +)/kg)	0.73 ± (0.06)a	0.61 ± (0.04)a	0.77 ± (0.06)a
CEC (cmol( +)/kg)	47.21 ± (1.36)a	34.89 ± (0.92)b	44.51 ± (1.96)a
Organic matter (%)	4.46 ± (0.60)a	3.35 ± (1.34)b	4.87 ± (0.10)a
Nitrogen (%)	0.23 ± (0.01)a	0.17 ± (0.02)b	0.26 ± (0.01)a
P (ppm)	17.18 ± (5.72)a	7.8 ± (0.73)b	17.64 ± (6.05)a
Dominant species in each plot	*Maytenus obscura Carissa edulis Olea* sp.	*Acacia abyssinica Buddleja polystachya Acacia nilotica*	*Albizia gummifera Prunus africana Brucea antidysenterica*
References	^83^	^85^	^84^

Values shown are means ± the standard errors of the means, which are indicated in parentheses. Values with different lowercase letters are significantly different (*p* < 0.05). The mean pH calculated from the [H+] and the mean+/− standard errors for pH are stated as 95% confidence intervals. The mean annual precipitation and mean annual temperature of each study area are based on nearby station data for 2019. asl, above sea level; CEC, cation exchange capacity; CRF30d: cumulative rainfall 30 days before sampling; EC, electrical conductivity. References for the climatic and geographical descriptions of the study areas are shown. To relate the vegetation characteristics to the fungal diversity, vegetation inventories were also conducted and the vegetation parameters determined. Plant parameters and their correlations with the fungal community were also used for further interpretation of the soil fungal pattern of each study area.

### Molecular analysis

A PowerSoil™ DNA Isolation Kit (MoBio Laboratories Inc., Carlsbad, CA, USA) was used to extract DNA from 0.25 g of soil per sample. PCR reactions of each sample were carried out in triplicate to minimize PCR biases. These triplicates were later pooled before sequencing. PCR reactions were performed in 20 μL reaction volumes containing 11.22 μL of modified quantization (MQ) water, 1.60 μL of DNA template, 2.00 μL of 10 × buffer, 1.40 μL of MgCl_2_ (50 mM), 1.60 μL of dNTPs (10 mM), 0.50 μL of bovine serum albumin (BSA) (2%), 0.80 μL of reverse and forward primers (10 μM), and 0.08 μL of Platinum Taq polymerase (Invitrogen, Carlsbad, CA, USA). The following PCR conditions were used: an initial denaturation step at 94 °C for 3 min; then 35 cycles at 94 °C for 45 s, 50 °C for 1 min, and 72 °C for 1.5 min; ending with one cycle at 72 °C for 10 min. To amplify the ITS2 rDNA region (ca. 260 bp), we used the forward primer fITS7^94^ and reverse primer ITS4^95^, appended with Illumina adaptors. To link the sequences to the sample source, a second PCR was performed to append sample-specific tags to the Illumina adaptors. The second PCR was conducted using Phusion HF PCR master mix and llumina indexes with the cycling conditions of 98 °C for 30 s, then followed by 6 cycles of denaturizing at 98 °C for 10 s, annealing at 55 °C for 30 s, elongating at 72 °C for 30 s, and a subsequent extension at 72 °C for 5 min and at 4 °C holds. Each set of PCR replicates also included a negative control comprising MQ water instead of DNA that underwent PCR under the same experimental conditions and was shown to be amplicon-free on a gel. The amplicon library was sequenced at BaseClear B.V. (Leiden, The Netherlands) using a paired-end (2 × 250 bp) Illumina MiSeq platform.

### Bioinformatic analysis

Primers and poor-quality ends in both directions (3′ and 5′) were removed based on a 0.02 error probability limit in Cutadapt v.2.8 with Phyton 3.6.7^96^. Because length differences in OTU clustering algorithms are often counted as terminal gaps that may result in clustering otherwise identical sequences into different OTUs, all sequences were truncated to 200 bp and then were filtered with USEARCH v.8.0^97^ to discard sequences with an expected error of > 1. The remaining sequences were collapsed into unique sequence types on a per-sample basis using USEARCH v.8.0^97^ while preserving read counts. First, we discarded singleton sequence types before grouping the remaining 105,840 high-quality sequences into 5152 OTUs with USEARCH at a 97% sequence similarity level while simultaneously excluding sequences representing OTUs with < 70% similarity or < 150 bp pairwise alignment length to a fungal sequence. Sequences were assigned to taxonomic groups based on pairwise similarity searches against the curated UNITE + INSD fungal ITS sequence database, version v.8.0 released on November 18th, 2018, which contains identified fungal sequences with assignments to species hypothesis (SH) groups^98^. Ecological functions at the genus level were assigned using the recent classification published^99^. We recognize the limitations of functional inference based on partial ITS sequences, and here use these guilds as hypothetical trophic groups. OTUs with > 90% similarity to a fungal SH with known ecological function were assigned either to plant pathogens, animal parasites, ectomycorrhizal (ECM) fungi, arbuscular mycorrhizal fungi, functional saprotrophs, or other groups. For genera that are known to comprise species from multiple trophic groups, their ecological function was assigned individually based on available ecological information for the matching SH in the UNITE database. A list of OTUs is provided as supplementary data to this article (Table S1).

### Vegetation sampling

To relate the vegetation characteristics to the soil fungal communities, vegetation inventories were conducted in each of the three forests in plots were established for soil and fungal sampling. Vascular plants identified in each plot were recorded using their vernacular names. For those species difficult to identify their scientific name in the field, specimens were collected and their taxonomic identification was conducted using the published volume of *The Flora of Ethiopia and Eritrea*^100^. Large trees growing outside the plots were included in the survey if their crowns overhung the plots because tree crown projection areas can affect macrofungal occurrence^101^. Furthermore, large trees create their own microhabitat and develop a large root system, providing more space for fungal associations^102^. Then, the vascular plant species richness and diversity parameters were determined (Table 1). Plant parameters and their correlations were also used for further interpretation of the soil fungal patterns of each study area. For calculating diversity and basal area of trees, data was collected on all woody plant species of greater than 5 cm diameter and found within 10 m distance from the smaller plots used for soil fungal sampling. In other words, vegetation sampling was done on a plot of 20 m* 50 m circumscribing the smaller plots (2 m*50 m) used for soil fungal sampling. This is because trees found within such distance are expected to affect fungal community composition in the smaller plots. The litter, roots, and shadow of these border trees enter the smaller plots and could affect fungal community composition and diversity. We also collected count data on vascular plants of less than 5 cm diameter, determined their density based on the number of individuals per plot which later converted to a per hectare basis. We related this data with fungal community composition and diversity.

### Statistical analysis

The data used for statistical analyses were transformed when needed to achieve the parametric criteria of normality and homoscedasticity. Fungal data were normalized by rarefying the abundance data to the smallest number of sequences per plot. In addition, soil variable data were scaled using base R. To compare fungal richness among forests, the relative richness of the fungal OTUs was calculated by dividing the number of OTU of the fungal group in question by the total number of all fungal OTUs on a per-sample basis^103^. Shannon’s H’ diversity index, H’ = –Σpi (lnpi)^104^, was estimated for each forest, where pi indicates the relative abundance of fungal species^105^. The Simpson’s diversity, D = 1–Σ(pi2), where pi is the importance probability in element I, and the Evenness, J = H´/H´max, where H´ is the number derived from the Shannon diversity index and the H´ max is the maximum possible value of H´, were also calculated^106^. The richness values for each forest were also estimated using the frequency of species detected during sampling to infer total species richness, often estimated from replicated samples of the community^107^. All diversity measures were analyzed using the Biodiversity R package^108^ in R version 4.0.3^109^.

Differences in soil, vegetation, and fungal variables across forests were assessed using linear mixed effects (LME) models^110^, where a block (plots in each forest area) was defined as random and a forest was defined as a fixed factor. LME models were used to prevent false-positive associations due to the relatedness structure in the sampling. A Tukey Test was used to test significant differences (*p* ≤ 0.05) between forests when needed. The relationship between fungi and vegetation attributes was determined through correlation analysis whenever needed. Variations among fungal trophic groups were analyzed separately.

Species accumulation curves were constructed to provide an estimate of soil fungal species richness in each forest. Curves were generated using Estimates Version 9^44^ and were based on total fungal OTU data sets with 1000 permutations. The diversity curves of the three church forests were also depicted using a Rényi diversity profile^111^, which depends upon the parameter alpha, such that alpha = 0 gives the total species number and alpha = 1 gives an index proportional to the Shannon index. To analyze correlations between the tree and fungal parameters, we performed a correlation matrix based on the Pearson Test using the Hmisc package. Cormat and pmat functions were also used to obtain and organize the results.

The relationship between soil fungal composition and the edaphic and vegetation parameters was visualized using non-metric multidimensional scaling (NMDS), based on a Hellinger-transformed OTU matrix, the fungal absent, and present data and environmental scaled data. The effects of forest were analyzed using a permutational multivariate ANOVA (PerMANOVA) based on 999 permutations using the adonis function in the vegan package. A one-way crossed analysis of similarities (ANOSIMs) was also performed to assess the significance of differences among groups observed in NMDS plots. Isolines of vascular plant richness were also plotted on NMDS ordinations using the ordisurf function. The strength of the difference between forests was measured by R values generated by ANOSIM, which take a value from 0 to 1, with 1 being the strongest possible difference^112^. Permutation tests using 999 replicates established the statistical significance (*p*) of the R values. A nonparametric test analysis was used because of the large number of zeros in the data set. The analysis was conducted using PAST software^113^. Effects of edaphic variables on the composition of the soil fungal community were determined based on the Bray–Curtis dissimilarity after excluding singleton OTUs. The correlation of NMDS axes scores with explanatory variables was assessed using the envfit function in R. To assess the influence of grouping edaphic, climate, location, and vegetation variables on the fungal community, we used the Mantel Test using Bray–Curtis distance on the total species matrix and Euclidean distance on environmental parameters.

## Supplementary Information

Supplementary Information 1.

Supplementary Information 2.

## Data Availability

Provisional Submission number NCBI (# 2523836).
