# Supplementary material for: Metabarcoding analysis of the soil fungal community to aid the conservation of underexplored church forests in Ethiopia
Source: Sci Rep. 2022 Mar 21;12:4817. doi: 10.1038/s41598-022-08828-3 (PMC8938458; doi:10.1038/s41598-022-08828-3)
Supplement: Supplementary file 1 — Supplementary Information 1. [file 41598_2022_8828_MOESM1_ESM.docx]

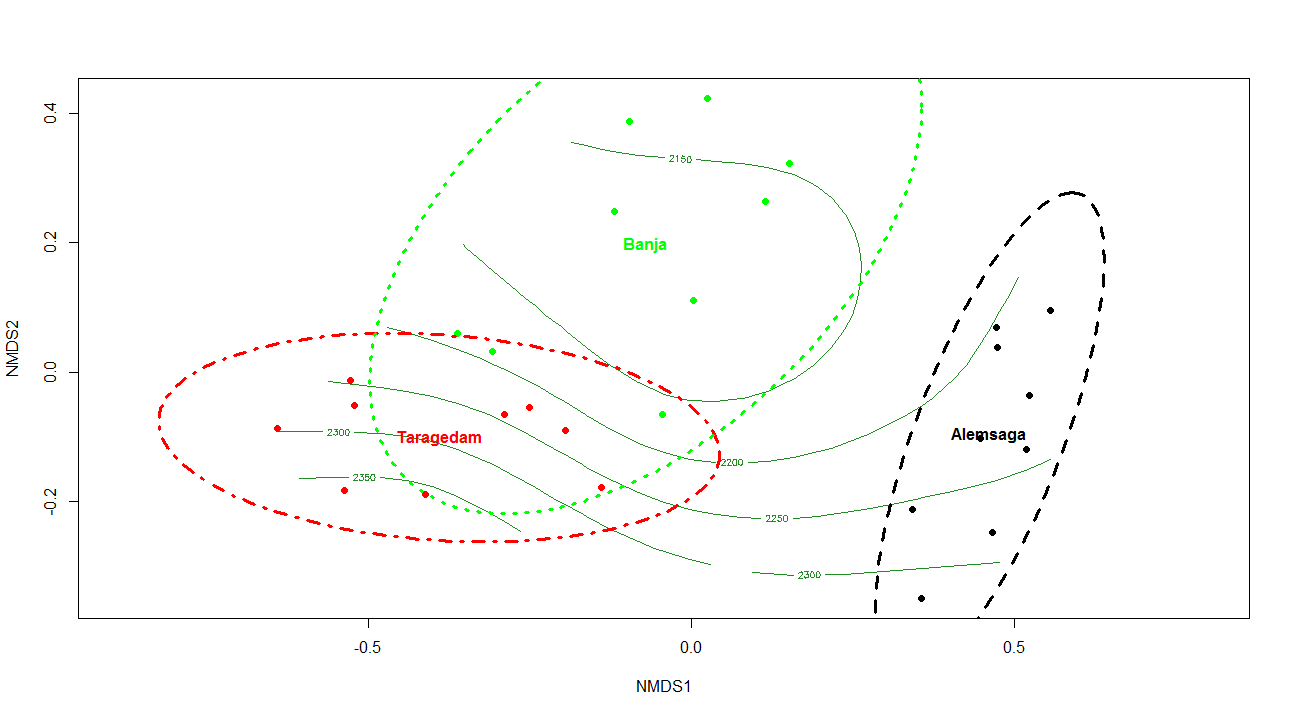


**Figure S1.** Non-metric multidimensional scaling (NMDS) ordination graph with fitted environmental variables based on dissimilarities calculated using the Bray–Curtis index of fungal community compositions of the three Dry Afromontane church forests in Northern Ethiopia with vascular tree richness displayed as isolines. The ordination was based on the absent and present fungal data. Ellipses indicate forest groups.
